# Predicting incident type 2 diabetes in a Japanese cohort: an 8-year analysis of the NAGALA database

**DOI:** 10.3389/fendo.2025.1465032

**Published:** 2025-05-15

**Authors:** Yangchun Wang, Fei Liu, Ruixiang Tong, Zhonghua He, Qin Fang, Jie Feng, Hongliang An, Junjun Liu

**Affiliations:** ^1^ Department of Endocrinology, Nanjing Meishan Hospital, Nanjing, China; ^2^ Department of General Surgery, Nanjing Meishan Hospital, Nanjing, China; ^3^ Department of Pharmacy, Nanjing Meishan Hospital, Nanjing, China; ^4^ Department of Psychiatry, Nanjing Meishan Hospital, Nanjing, China

**Keywords:** type 2 diabetes mellitus, incidence, predictive markers, cohort study, Japanese population

## Abstract

**Objective:**

Using data from the NAGALA database, this retrospective cohort study set out to identify the predictive markers for incident Type 2 Diabetes Mellitus (T2DM), with a particular focus on the non-diabetic Japanese population.

**Methods:**

We examined the data from a cohort of 15,464 individuals (with a male representation of 54.5% and an average age of (43.71 ± 8.90 years) sourced from the NAGALA (NAfld in the Gifu Area Longitudinal Analysis) research which was a longitudinal study at Medical Health Checkup Center of Murakami Memorial Hospital. The analysis focused on the incidence of T2DM from 2004 to 2012. Baseline demographic, anthropometric, biochemical, and lifestyle data were collected. All participants were not type 2 diabetic at baseline. The diagnosis of T2DM was confirmed by HbA1c >= 48mmol/mol, fasting plasma glucose >= 126mg/dL, or diabetes reported by oneself. Multivariate analysis was performed after univariate Cox regression analysis was used to find early determinants of T2DM incidence. The ability of individual components and a composite risk score to discriminate was assessed using Receiver Operating Characteristic (ROC) curves.

**Results:**

Over an average follow-up duration of 2207.82 ± 1379.73 days, 262 patients (1.7%) had the onset of T2DM. Following the removal of confounding variables we found that age (*HR*=1.03,95%*CI* 1.01∼1.04, *P*=0.001), waist circumference(*HR*=1.05,95%*CI* 1.03∼1.06, *P*<0.001), alanine transaminase(*HR*=1.01,95%*CI* 1.00∼1.01, *P*=0.045), glycated hemoglobin (HbA1c) (*HR*=24.30,95%*CI* 15.69∼37.63,*P*<0.001), fasting plasma glucose(*HR*=1.10,95%*CI* 1.07∼1.12, *P*<0.001), the presence of fatty liver(*HR*=1.86,95%*CI* 1.37∼2.53, *P*<0.001), current smoking(*HR*=1.61,95%*CI* 1.16∼2.23, *P*=0.004), and heavy alcohol consumption(*HR*=1.79,95%*CI* 1.06∼2.99, *P*=0.028) were identified as independent risk factors for T2DM(all *P* < 0.05), while high-density lipoprotein cholesterol (HDL-C) exhibited a protective effect (*HR=*0.98,95%*CI* 0.97∼1.00*, P=*0.010). The area under the Receiver Operating Characteristic (ROC) curve for individual factors ranged from 0.53 to 0.83, with the highest value for HbA1c. A combined risk model incorporating these factors including age, waist circumference, alanine transaminase, HbA1c, fasting plasma glucose, the presence of fatty liver, current smoking, heavy alcohol consumption, 1/HDL-C achieved an AUC of 0.90 (95% *CI* 0.88-0.92, *P* < 0.001), signifying robust discriminatory ability. At a predictive probability threshold of >0.017, the model exhibited sensitivity and specificity of 0.863 and 0.828, respectively.

**Conclusion:**

Current research has underscored the significance of a multifaceted approach to the prevention of T2DM, which includes early intervention targeting modifiable risk factors such as obesity, unhealthy alcohol use, and smoking, in conjunction with the monitoring of key metabolic markers like HbA1c and liver enzymes.

## 1 Introduction

In the 21st century, the development of Type 2 Diabetes Mellitus (T2DM) has emerged as a significant global medical challenge, imposing substantial burdens on health systems and economies worldwide (1). The latest iteration of the International Diabetes Federation’s Diabetes Atlas highlights a stark increase in diabetes prevalence, with nearly half a billion individuals affected globally, the majority of whom have T2DM (2). Characterized by hyperglycemia resulting from impaired insulin action and inadequate insulin production (3), this chronic condition is intricately linked to a wide range of debilitating complications, including nephropathy, cardiovascular diseases, retinopathy, and neuropathy, which profoundly impact both quality of life and life expectancy (4).

Several salient features characterize the evolving trend of T2DM. Firstly, a notable trend of increasing diagnoses among children and adolescents is observed, closely linked to the rising obesity epidemic (5). The important contribution of obesity to early onset T2DM in the younger generations has well been shown in an early population-based study conducted in the Chinese ethnicity living in Taiwan (6). This highlights the long-term detrimental health consequences of suboptimal dietary patterns and sedentary lifestyles. Secondly, socio-economic disparities play a pivotal role in shaping the disease burden, with individuals from lower education and income brackets disproportionately affected, reflecting the entrenched issue of health inequity (7). Lastly, while technological advancements and medical breakthroughs have improved the efficacy of disease management, they have also contributed to rising treatment costs, presenting new challenges for public health policy formulation and implementation (8).

Multiple factors have been identified as potential contributors to the risk of developing T2DM. A comprehensive meta-analysis has highlighted a wide array of confounding variables, including genetically predisposed low basal metabolic rate (BMR), excess weight and obesity, sedentary lifestyles, dietary patterns, advancing age, ethnic background, inadequate sleep duration, obstructive sleep apnea, psychosocial stressors, and alterations in gut microbiota, all of which have been associated with an increased risk of T2DM (9–12). Moreover, exposures to arsenic (13), dioxin (14), polychlorinated biphenyls (15), and several other toxicants (16) have been extensively researched and proved to be important diabetogenic factors. Notably, however, the cumulative impact of these various risk factors remains underexplored, with a lack of studies investigating their combined effects on the onset and progression of the disease.

The current investigation distinguishes itself by adopting an extensive eight-year longitudinal perspective, which captures the nuanced progression of T2DM from initial susceptibility to clinical diagnosis while challenging conventional temporality limits. Unlike prior studies, our approach emphasizes the dynamic interplay of risk factors over time, ensuring a more accurate representation of disease development. We innovatively integrate cutting-edge epidemiological knowledge within a robust methodological framework, allowing for a novel examination of not only cumulative impacts but also the complex, potentially synergistic interactions among various factors such as obesity, exercise routines, smoking, alcohol use, hypertension, dyslipidemia, liver dysfunction, and glycemic imbalances, among others. By employing sophisticated statistical methodologies, we aim to decipher these intricate relationships, ultimately identifying distinct high-risk profiles that can inform precision prevention strategies. This meticulous study aspires to shed new light on the etiology of T2DM, fostering the design of targeted interventions and personalized preventive measures, thereby enhancing opportunities for early detection and intervention.

## 2 Materials and methods

### 2.1 Data source

The raw data used in this investigation were collected from the dataset provided by Okamura et al., which is accessible through the DATADRYAD repository (http://datadryad.org). We ensure compliance with Dryad’s Terms of Service by appropriately citing the Dryad dataset (17) within our work.

### 2.2 Data description

The dataset encompasses baseline demographics, cases of new-onset T2DM at follow-up, and follow-up durations. Extracted variables include T2DM incidence, follow-up duration, age, gender, body mass index (BMI), waist circumference (WC), presence of fatty liver, smoking status, alcohol consumption categories, exercise habits, and various biochemical markers such as alanine aminotransferase (ALT), aspartate transaminase (AST), gamma-glutamyl transferase (GGT), high-density lipoprotein cholesterol (HDL-C), total cholesterol (TC), triglycerides (TG), hemoglobin A1c (HbA1c), fasting plasma glucose (FPG), diastolic blood pressure (DBP), and systolic blood pressure (SBP).

### 2.3 Design of the study and subjects

**Figure 1:**
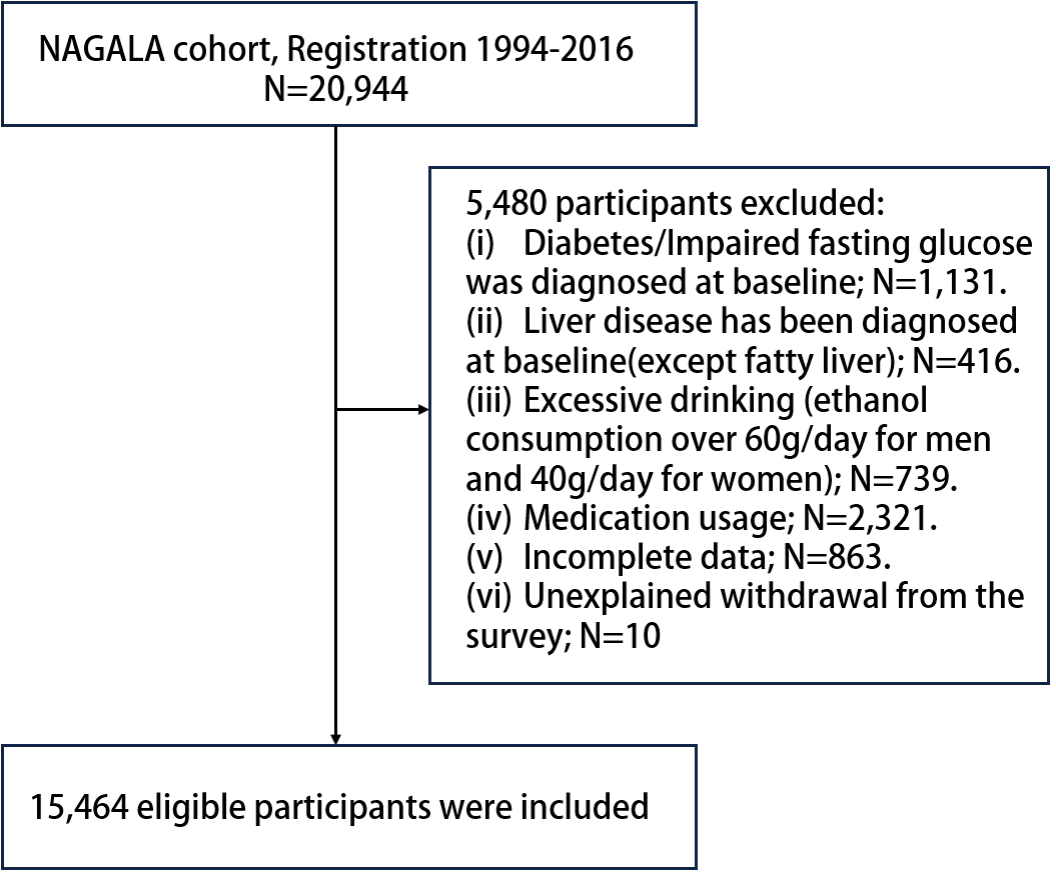
Flowchart of study participants.

Utilizing the NAGALA (NAfld in the Gifu Area, Longitudinal Analysis) system, Okamura et al. (17) initially explored the influence of obesity phenotypes on the risk of developing T2DM. Due to the need for repeated assessments, a longitudinal follow-up was conducted to ascertain incident T2DM through laboratory tests and abdominal ultrasounds (18) which were employed to determine the presence of fatty liver (19). Our work represents a secondary examination of the publicly available NAGALA dataset. At the baseline in 2004, the study population was free of T2DM. Then, regular (annual) follow - up was carried out for a total of eight years until 2012, aiming to observe the incidence rate and related factors of T2DM in this part of the population. The inclusion criteria for participants were thoroughly detailed in the original publication (20). In this study, based on the new research hypothesis, we selected the target population of the current study according to the following exclusion criteria: (a) subjects with alcohol abuse (21) (>60 g per day for men and >40 g per day for women); (b) subjects with diabetes/impaired fasting glucose [6.1 mmol/L< baseline FPG <7.8 mmol/L (22)]/liver disease (except fatty liver) at baseline; (c) subjects who were taking medication at baseline; (d) subjects with incomplete physical examination data; (e) subjects who withdrew from the survey for unknown reasons. Finally, we included 15,464 eligible subjects, and **Figure 1** shows the process of inclusion and exclusion of the study population.

### 2.4 Data collection and measurements

Participants completed a standardized questionnaire regarding medical histories and lifestyle factors, including exercise frequency, alcohol intake, and smoking behavior. Alcohol consumption was quantified based on self-reported weekly averages over the past month, categorizing participants as non/light drinkers (21) (<280g/week for males, <140g/week for females), moderate drinkers (280-420g/week for males, 140-280g/week for females), or heavy drinkers (21) (>420g/week for males, >280g/week for females). Smoking habits classified individuals as never, past, or current smokers, with definitions provided in the text. Participating in any kind of physical activity at least once a week was considered regular exercise. Clinical information on a participant’s health check-up was composed of standardized and uniform questionnaire records used by trained medical staff, including SBP, DBP, age, weight, sex, WC, smoking status, and exercise habits. Laboratory test indicators included TG, HbA1c, HDL-C, FPG, TC, ALT, AST, and GGT were measured using an automatic biochemistry analyzer.

### 2.5 Definitions

T2DM diagnosis was confirmed by HbA1c >= 48mmol/mol, fasting plasma glucose >= 126mg/dL, or diabetes reported by oneself at the time of the follow-up (22).

### 2.6 Ethical approval

Given this study’s nature as a secondary analysis of de-identified, pre-existing data, no additional participant consent was necessary. Ethical clearance for the original study and data sharing is documented in the referenced publication (17). The research received approval from Peking University Third Hospital’s Medical Ethics Committee (approval number:M2018149).

### 2.7 Outcomes

All participants underwent regular physical check-ups at Murakami Memorial Hospital, and 60% of the individuals underwent check-ups once or twice a year (17). Event T2DM was defined as the occurrence of at least one of the following conditions during follow-up: self-reported diagnosis of T2DM, FPG level >=7 mmol/L, or HbA1c level >= 6.5% (22).

### 2.8 Statistical analyses

Version 26.0 of IBM SPSS Statistics was used to analyze data. In order to evaluate the normality of the variance distribution of continuous characteristics, the Kolmogorov-Smirnov test was utilized. Variables conforming to normal distribution were summarized as means ± standard deviations (SD), while those deviating from normality were described using medians accompanied by interquartile ranges (IQR). Categorical data were presented as frequencies and respective percentages (%). Initially, univariate proportional hazards Cox regression models were used to look at the specific relationships between each predictor and the likelihood of acquiringT2DM. The following variables were included in the univariate analysis: age, sex, WC, BMI, ALT, AST, GGT, TC, HDL-C, TG, HbA1c, FPG, SBP, DBP, Fatty liver, Habit of exercise, Smoking status, Alcohol consumption. The Wald test was utilized to assess the statistical importance after calculating the probability ratios (HRs) and their confidence intervals of 95% (CIs). In the univariate analysis, predictors with P-values under 0.1 moved on to the multivariate phase. To mitigate multicollinearity issues, variables with a variance inflation factor (VIF) exceeding 5 were omitted from the final multivariate Cox model. This model was then employed to derive adjusted HRs and their corresponding 95% CIs for incident T2DM. The discriminatory power of different parameters in predicting the incidence of T2DM was also determined through the use of receiver operating characteristic (ROC) curve analysis. After identifying the significant risk factors from the Cox regression analysis, we then incorporated all these risk factors into a multiple logistic regression model. The discriminatory power of different parameters in predicting the incidence of T2DM was also determined through the use of receiver operating characteristic (ROC) curve analysis. ROC curves were generated for both the significant risk factors identified in the multivariate analysis and for the combined effect of all risk factors. An area under the curve (AUC) between 0.7 and 0.8 was considered appropriate for practical discrimination. Optimal cutoff points for continuous variables were determined by maximizing the Youden index (J = sensitivity + specificity - 1) from ROC curve analysis. Statistical significance was indicated throughout the investigation using a two-tailed P-value threshold of 0.05.

## 3 Results

### 3.1 Demographic and clinical characteristics of subjects

The study included 15,464 individuals, with a gender split of 54.5% males to 45.5% females, averaging 43.71 ± 8.90 years old. Baseline demographics and clinical measures are in **Table 1**. The mean BMI was 22.13 ± 3.11 kg/m²; waist circumference, 76.47 ± 9.11 cm. Liver enzymes (ALT, AST, GGT) were 19.99 ± 14.34 IU/L, 18.40 ± 8.64 IU/L, and 20.31 ± 18.14 IU/L, respectively. Lipid profiles showed TC at 198.20 ± 33.41 mg/dL, HDL at 56.54 ± 15.57 mg/dL, and TG at 80.79 ± 58.05 mg/dL. Glucose indicators HbA1c and FPG averaged 5.17 ± 0.32% and 92.97 ± 7.44 mg/dL. Mean blood pressures: SBP 114.50 ± 14.97 mmHg; DBP 71.58 ± 10.50 mmHg. Fatty liver prevalence was 17.7%. Moreover, 82.5% lacked exercise routines, 58.4% didn’t smoke, and 76.3% avoided alcohol.

**Table 1:** Participants characteristics at baseline (N=15464).

Variables	MEAN±SD OR N (%)
Age, years	43.71±8.90
WC, cm	76.47±9.11
BMI, kg/m^2^	22.12±3.13
ALT, IU/L	19.99±14.34
AST, IU/L	18.40±8.64
GGT, IU/L	20.31±18.14
TC, mg/dl	198.20±33.41
HDL, mg/dl	56.54±15.57
TG, mg/dl	80.79±58.05
HbA1c, %	5.17±0.32
FPG, mg/dl	92.97±7.44
SBP, mmHg	114.50±14.97
DBP, mmHg	71.58±10.50
Sex	
Female	7034 (45.5)
Male	8430 (54.5)
Fatty liver	
No	12723 (82.3)
Yes	2741 (17.7)
Habit of exercise	
No	12755 (82.5)
Yes	2709 (17.5)
Smoking status	
Never	9031 (58.4)
Past	2952 (19.1)
Current	3481 (22.5)
Alcohol consumption	
None	11805 (76.3)
Light	1758 (11.4)
Moderate	1360 (8.8)
Heavy	541 (3.5)

Mean and SD are shown for the continuous variables (Age; WC; BMI; ALT; AST; GGT; TC; HDL; TG; HbA1c; FPG; SBP; DBP). N and % are shown for the categorical variables (sex; fatty liver; Habit of exercise; Smoking status; Alcohol consumption). WC, waist circumference; BMI, body mass index; AST, aminotransferase; ALT, aminotransferase; GGT, gamma-glutamyl transferase; TC, total cholesterol; HDL-C, high-density lipoprotein cholesterol; TG, triglyceride; HbA1c, hemoglobin A1c; FPG, fasting plasma glucose; SBP, systolic blood pressure; DBP, diastolic blood pressure.

### 3.2 Univariate analysis of factors associated with T2DM incidence

The follow-up time for this study was (2207.82±1379.73) days. 262 participants (1.7%) were diagnosed with T2DM. The incidence density was approximately 2.12 per 1,000 person-years. Cox regression analysis was used with 8 years of diabetes as the dependent variable and baseline information as the independent variable, taking into account follow-up time. The results of the one-way COX regression analysis showed HDL was negatively associated with the development of T2DM (HR=0.94,95%CI 0.93∼0.95*,P*<0.001), but age(HR=1.06,95%CI 1.04∼1.07*,P*<0.001), WC(HR=1.10,95%CI 1.09∼1.11*,P*<0.001), BMI(HR=1.25,95%CI 1.23∼1.28*,P*<0.001), AST(HR=1.01,95%CI 1.00∼1.01*,P*<0.001), ALT(HR=1.01,95%CI 1.00∼1.01*,P*<0.001), TC(HR=1.01,95%CI 1.00∼1.01*,P*<0.001), GGT(HR=1.01,95%CI 1.00∼1.01*,P*<0.001), TG(HR=1.01,95%CI 1.00∼1.01,P<0.001), FPG(HR=1.21,95%CI 1.12∼1.23*,P*<0.001), HbA1c(HR=83.57,95%CI 56.92∼122.70*,P*<0.001), DBP(HR=1.05,95%CI 1.04∼1.06*,P*<0.001), SBP(HR=1.03,95%CI 1.03∼1.04*,P*<0.001), male(HR=2.83,95%CI 2.11∼3.79*,P*<0.001), fatty liver(HR=7.86,95%CI 6.12∼10.10*,P*<0.001) were strongly correlated with the occurrence of T2DM,all P<0.001. Using the never-smoking group as the reference group, the past-smoking group(HR=1.62,95%CI 1.15∼2.26*,P*<0.001) and the current-smoking group(HR=2.87,95%CI 2.19∼3.76*,P*<0.001) were positively correlated with the incidence of T2DM. Compared to the never-drinking group, the light alcohol consumption group (HR=0.88, 95% CI 0.59–1.33, P<0.001) exhibited a negative correlation with the incidence of T2DM. Conversely, the heavy alcohol consumption group (HR=2.21, 95% CI 1.39–3.50, P<0.001) showed a positive association with the occurrence of T2DM. Meanwhile, the moderate alcohol consumption group displayed no significant correlation with the 8-year incidence of diabetes. There was no correlation between having an exercise habit and the incidence of T2DM compared to those who did not have an exercise habit (HR=0.90, 95% CI 0.64–1.25, P=0.525).

### 3.3 Multivariate analysis of independent risk factors for T2DM

The final multivariate Cox regression model for T2DM includes variables of p-values <0.1 in univariate tests, such as age, WC, BMI, AST, ALT, TC, GGT, HDL, FPG, TG, HbA1c, DBP, SBP, sex, smoking, fatty liver, and alcohol intake. To address potential multicollinearity, we conducted a VIF analysis. Variables with a VIF greater than 5 were excluded from the final multivariate Cox model. Based on this criterion, the following variables were excluded from the final model: BMI, AST, and DBP. The exclusion of these variables did not significantly alter the overall results of the model. The results of the regressions are illustrated in **Table 2**. After adjusting for confounding factors we found that age(HR=1.03,95%CI 1.01∼1.04,P=0.001),WC (HR=1.05,95%CI 1.03∼1.06,P<0.001),ALT(HR=1.01,95%CI 1.00∼1.01,P=0.045),HbA1c(HR=24.30,95%CI 15.69∼37.63,P<0.001),FPG(HR=1.10,95%CI 1.07∼1.12,P<0.001), fatty liver(HR=1.86,95%CI 1.37∼2.53,P<0.001), were independent risk factors of T2DM.Current smoking group was positively associated with incidence of T2DM compared to Never Smoking Group (HR=1.61,95%CI 1.16∼2.23,P=0.004). Heavy alcohol consumption (HR=1.79,95%CI 1.06∼2.99, P=0.028) was independent risk factors of T2DM, using the never drinking group as reference. In the contrary, HDL (HR=0.98,95%CI 0.97∼1.00, P=0.010) was independent protective factors of T2DM. (**Table 2**).

**Table 2:** Result of univariate and multivariate analysis and implications for the development of type 2 diabetes.

Variables	Univariate analysis			Multivariate analysis		
	HR	*95%CI*	*P value*	HR	*95%CI*	*P value*
Age, years	1.06	1.04∼1.07	<0.001	1.03	1.01∼1.04	0.001
WC, cm	1.10	1.09∼1.11	<0.001	1.05	1.03∼1.06	<0.001
BMI, kg/m^2^	1.25	1.23∼1.28	<0.001	–	–	–
ALT, IU/L	1.01	1.00∼1.01	<0.001	1.01	1.00∼1.01	0.045
AST, IU/L	1.01	1.00∼1.01	<0.001	–	–	–
GGT, IU/L	1.01	1.00∼1.01	<0.001	1.00	1.00∼1.01	0.349
TC, mg/dl	1.01	1.00∼1.01	<0.001	1.00	0.99∼1.00	0.220
HDL, mg/dl	0.94	0.93∼0.95	<0.001	0.98	0.97∼1.00	0.010
TG, mg/dl	1.01	1.00∼1.01	<0.001	1.00	1.00∼1.01	0.123
HbA1c, %	83.57	56.92∼122.70	<0.001	24.30	15.69∼37.63	<0.001
FPG, mg/dl	1.21	1.12∼1.23	<0.001	1.10	1.07∼1.12	<0.001
SBP, mmHg	1.03	1.03∼1.04	<0.001	0.99	0.98∼1.00	0.198
DBP, mmHg	1.05	1.04∼1.06	<0.001	–	–	–
Sex						
Female	1.00 (Reference)			1.00 (Reference)		
male	2.83	2.11∼3.79	<0.001	0.70	0.47∼1.03	0.071
Fatty liver						
No	1.00 (Reference)			1.00 (Reference)		
Yes	7.86	6.12∼10.10	<0.001	1.86	1.37∼2.53	<0.001
Habit of exercise						
No	1.00 (Reference)			–	–	–
Yes	0.90	0.64∼1.25	0.525	–	–	–
Smoking status						
Never	1.00 (Reference)			1.00 (Reference)		
Past	1.62	1.15∼2.26	<0.001	0.79	0.54∼1.17	0.241
Current	2.87	2.19∼3.76	<0.001	1.61	1.16∼2.23	0.004
Alcohol consumption						
No	1.00 (Reference)			1.00 (Reference)		
Light	0.88	0.59∼1.33	<0.001	1.09	0.71∼1.67	0.704
Moderate	1.29	0.87∼1.92	0.212	1.28	0.83∼1.97	0.257
Heavy	2.21	1.39∼3.50	0.001	1.79	1.06∼2.99	0.028

Abbreviations as in **Table 1**.

### 3.4 ROC analysis for predictive values

**Figure 2:**
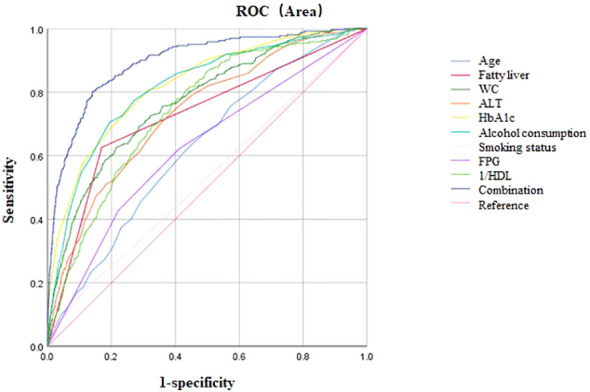
ROC curve analysis. Area under the curve of Age, Fatty liver, WC, ALT, HbA1c, Alcohol consumption, Smoking status; FPG; HDL; Combination; Reference.

AUC for ROC analysis revealed individual discriminatory power as follows: age (0.63), fatty liver (0.73), WC (0.77), ALT (0.74), HbA1c (0.83), heavy alcohol consumption (0.53), current smoking (0.62), FPG (0.82), and inverse HDL (0.74). When these characteristics were combined, the AUC increased to 0.90 (*P*< 0.001, 95%*CI* 0.88–0.92), indicating better predictive ability for T2DM (**Table 3**, **Figure 2**). The Youden index peak occurred at a predictive probability threshold of >0.02, with corresponding sensitivity and specificity of 0.863 and 0.828, respectively.

**Table 3:** Area under the curve of Age, Fatty liver, WC, ALT, HaA1c, Alcohol consumption, Smoking status; FPG; 1/HDL; Combination.

Variables	AUC (95%*CI*)	*P value*	Cutoff Point	Sensitivity	Specificity
Age	0.63 (0.59∼0.66)	<0.001	40.50	0.759	0.430
Fatty liver	0.73 (0.69∼0.76)	<0.001	–	–	–
WC	0.77 (0.74∼0.80)	<0.001	81.05	0.654	0.716
ALT	0.74 (0.71∼0.77)	<0.001	18.50	0.745	0.600
HbA1c	0.83 (0.80∼0.85)	<0.001	5.38	0.743	0.689
Alcohol consumption	0.53 (0.49∼0.57)	0.102	–	–	–
Smoking status	0.62 (0.59∼0.66)	<0.001	–	–	–
FPG	0.82 (0.79∼0.84)	<0.001	5.52	0.673	0.808
1/HDL	0.74 (0.71∼0.77)	<0.001	0.78	0.710	0.639
Combination	0.90 (0.88∼0.92)	<0.001	0.02	0.863	0.828

Abbreviations as in **Table 1**; AUC, Area under the curve.

## 4 Discussion

In the present investigation, we meticulously examined the 8-year incidence of T2DM and its determinants within a Japanese cohort. The primary objective was to elucidate the factors contributing to the onset of T2DM. Our findings revealed that (1) during an average follow-up period of 2207.82 ± 1379.73 days, 262 participants (1.7%) acquired T2DM; (2) age, WC, ALT, HbA1c, FPG, the presence of fatty liver, current smoking status, and heavy alcohol intake were identified as separate risk variables, whereas HDL-C exhibited a shielding effect; (3) elevated HbA1c levels were correlated with an increased susceptibility to T2DM. The most potent predictor of 8-year T2DM incidence was a combination of age, fatty liver status, WC, ALT, HbA1c, heavy alcohol consumption, current smoking, FPG, and inverse HDL-C (area under the curve, AUC = 0.90).

During an average follow-up time of 2207.82 ± 1379.73 days, 262 participants (1.7%) acquired T2DM. This seems to be too low and may be a big challenge to the validity of the dataset that the investigators were using. In the Chinese ethnicity living in Taiwan, the calculated incidence based on oral glucose tolerance tests (OGTT) was approximately 1% to 4% per year (23). Similar incidence was consistently reported (9). The inconsistency between our findings and previous studies may be attributable to several factors. Firstly, differences in the diagnostic criteria and methods used to identify T2DM across studies could contribute to the discrepant results. While our study relied solely on blood glucose and HbA1c levels to diagnose T2DM, other investigations may have incorporated additional diagnostic tests, such as OGTT or diabetes-specific autoantibody screening, which could lead to the identification of different patient populations. Secondly, the characteristics of the study samples may have varied across the existing literature. Differences in factors such as age, ethnicity, comorbidities, and disease severity among the participants enrolled could potentially influence the observed incidence or prevalence of T2DM, thereby accounting for the divergent findings. It is important to note that these potential explanations for the inconsistency are speculative and would require further investigation to confirm.

The correlation we observed between advanced age and an increased chance of T2DM development is in harmony with the body of established research, echoing findings from similar studies (6, 24, 25). This association can be explained by a host of pathophysiological changes inherently linked to the aging process. Primary among these is the age-related decline in insulin sensitivity, an essential component in the emergence of insulin resistance, a quintessential feature of T2DM, which is known to intensify with advancing years (26). This phenomenon can be partly attributed to changes in adipose tissue distribution and functionality. With aging, there is a propensity toward central obesity, which is intrinsically connected to insulin resistance (27). The dysfunction of adipose tissue, typified by an increase in pro-inflammatory cytokine secretion and a decrease in adiponectin levels, further aggravates insulin resistance (28). In addition, the functionality of pancreatic β-cells tends to wane with age (29). The β-cells’ capacity to emit adequate insulin in response to glucose diminishes over the years, which impairs glucose tolerance and can ultimately result in T2DM (30). This deterioration in β-cell function may stem from oxidative stress, persistent inflammation, and a lessened capacity for regeneration (31). Moreover, aging is accompanied by a decrease in physical activity and a loss of muscle mass, both of which significantly influence insulin sensitivity. Lower levels of physical activity result in reduced glucose uptake by muscles, while sarcopenia, the age-related loss of muscle mass, diminishes the body’s ability to dispose of glucose in response to insulin stimulation (32). Finally, the aging process is attended by a state of low-grade chronic inflammation, termed “inflammaging” (33). This persistent inflammatory condition contributes to the emergence of insulin resistance through a variety of mechanisms, including the activation of stress kinases that disrupt normal insulin signaling (31). Elucidating these mechanisms is vital for crafting targeted interventions intended to prevent or postpone the onset of T2DM in the elderly population.

Blood pressure is highly related to obesity (34) and is one of the components of the metabolic syndrome. Hypertension and diabetes mellitus are mutually corelated (35). However, our analysis revealed a lack of significant association between SBP and incident T2DM, contrasting with findings from other studies (36, 37). A mendelian randomization analysis by Sun et al. contributed to providing a conclusion: T2DM may causally affect hypertension, whereas the relationship from hypertension to T2DM is unlikely to be causal (38). This suggests that while there may be a bi-directional relationship between these two conditions, the direction of causality is not symmetric. Our findings, therefore, emphasize the need for further research to elucidate the underlying mechanisms and pathways through which T2DM and hypertension interact. Additionally, these results highlight the importance of considering the complexity of the pathophysiological processes when designing interventions and treatment strategies for patients with T2DM and hypertension.

T2DM, hypertension and dyslipidemia are among the 3 main metabolic diseases that may result from obesity (39). Except for FPB and HbA1c, WC seems to be a simple, easy, modifiable and most predictive parameter than any other single factor. Contrary to FPB and HbA1c, WC is surely important predictors for T2DM because they are parameters representing the internal milieu related to glucose metabolism and T2DM but the data can only be collected with blood samples taken. The measurement of WC, indicative of abdominal obesity, is robustly linked to an augmented risk of T2DM onset, in alignment with the findings of a comprehensive cross-sectional study conducted in China (40). The relationship between WC and T2DM is complex, involving a spectrum of physiological and biochemical changes that culminate in insulin resistance and dysregulated glucose metabolism (41). The unique metabolic characteristics of visceral abdominal fat tissue (VAT) adipocytes may be explained by the increased expression of endocrine receptors (androgen, adrenergic, estrogen, and glucocorticoid) at the cellular level (42). Moreover, VAT adipocytes exhibit reduced adipogenic potential compared to their subcutaneous adipose tissue (SCAT) counterparts, leading to dysregulated cellular hypertrophy that outstrips the existing vascular supply, causing oxygen deprivation and ultimately necrosis caused by ischemia. Adipose tissue is invaded by inflammatory macrophages, causing fibrotic alterations and a persistent, low-grade inflammatory state as the tissue responds to chronic hypoxia and cell death (43). This environment prompts paracrine signaling within the surrounding adipocytes, resulting in heightened insulin resistance, diminished glucose and free fatty acid (FFA) uptake, and an increase in lipid oxidation (42). Additionally, since SCAT is thought to act as a metabolic sink or buffer for TGs and FFAs, a higher VAT: SCAT ratio is linked to a risky metabolic profile (44). Furthermore, aberrant adipokines are exposed to the liver by the arterial drainage of visceral fat straight into the portal circulation, which modifies cerebral metabolism of lipids and encourages fatty deposits (45). Collectively, these VAT-related characteristics likely underpin the increased susceptibility to diabetes observed in individuals with visceral obesity.

Our findings highlight the role of ALT as a separate risk factor for the development of T2DM during an 8-year monitoring period. Elevated ALT levels in those who subsequently develop T2DM may indicate the existence of a condition called non-alcoholic fatty liver disease (NAFLD), a disorder closely associated with metabolic syndrome and insulin resistance. Notably, our analysis suggests that hepatic fat content is a significant contributor to the onset of T2DM. NAFLD is defined by the excessive accumulation of triglycerides within hepatocytes, which can disrupt glucose metabolism and facilitate the development of T2DM (46). The liver is essential in preserving glucose homeostasis, and hepatic insulin resistance can result in heightened glucose production and diminished insulin clearance—both crucial elements in the pathogenesis of T2DM (47). Treatments for T2DM and NAFLD were the subject of a network meta-analysis and systematic review, which revealed a strong correlation between the two disorders and how each accelerated the development of the other. The study’s therapeutic strategies underscored the significance of ALT levels, underscoring their importance in the management of both T2DM and NAFLD (48). These results suggest that ALT levels are an important marker and are crucial for the diagnosis and treatment of T2DM.

The data presented confirm the pivotal role of HbA1c and FPG as independent prognosticators of incident T2DM across an 8-year follow-up. The robust correlation between elevated HbA1c and FPG levels and the onset of T2DM highlights the utility of these biomarkers in pinpointing individuals with a heightened risk of developing the disease. HbA1c, an indicator of prolonged glycemic control, is broadly acknowledged as a reliable forecaster of T2DM. In our investigation, HbA1c exhibited the largest area under the ROC curve among the individual factors, signifying its substantial ability to discern T2DM risk. HbA1c is a vital tool for identifying people with impaired glucose management who may be at risk of developing T2DM since it can reflect mean levels of blood glucose over the previous two to three months. FPG, a conventional index of glucose homeostasis, also proved to be a significant risk factor for T2DM in our analysis. Elevations in FPG signify deteriorated β cell function and insulin resistance, which are fundamental to the pathogenesis of T2DM. The uniformity of FPG as a predictive marker across diverse studies emphasizes its clinical relevance for both risk evaluation and diabetes screening (49). A bidirectional cohort study among a Thai population with impaired fasting glucose (IFG) revealed that individuals with IFG and HbA1c abnormalities had a greater likelihood of developing diabetes than those with IFG alone, suggesting that the conjunction of HbA1c and FPG may delineate a high-risk subset of IFG patients for T2DM (50). Routine monitoring of these biomarkers in clinical settings is imperative for the early detection of high-risk individuals and for the implementation of preventive strategies aimed at curbing the incidence of T2DM.

During an 8-year follow-up period, our analysis showed that current smoking status emerged as a separate risk component for the development of T2DM. This observation is in concordance with an extensive body of prior research that has uniformly identified smoking as a considerable predictor of incident T2DM (51). Arsenic exposure has been shown to be associated with T2DM (52) and cigarettes contain arsenic. Smoking not only elevates the risk of developing T2DM but may also exacerbate the risk of complications among those already diagnosed with the condition (53). A nationwide population-based cohort study originating from South Korea has illuminated that smoking cessation can effectively mitigate the risk of diabetes. However, it is worthy of note that a dose-dependent relationship may exist between smoking intensity and diabetes development. Even following smoking cessation, the risk of diabetes persists among individuals with a history of heavy and extreme smoking. These insights emphasize the critical importance of reducing smoking and minimizing secondhand smoke exposure, particularly among individuals striving to prevent and manage T2DM.

The present study identified heavy alcohol consumption as a distinct risk factor for the onset of T2DM across an 8-year follow-up period. A retrospective cohort study among Japanese males has independently linked heavy drinking to an elevated risk of incident T2DM (54). Furthermore, studies have indicated that not drinking alcohol may also be linked to a higher chance of developing T2DM (55). These findings reinforce the connection between heavy drinking and a heightened risk of developing T2DM, a relationship that appears to be more pronounced in men. Consequently, these data underscore the significance of moderating alcohol intake, particularly among individuals at risk for T2DM.

The identification of HDL-C as a protective factor against T2DM, in spite of conflicting evidence within the literature, warrants deeper investigation into its multifaceted role in the prevention of diabetes. The protective effect seen in this study is consistent with results from a Chinese study that examined the link between levels of HDL-C and the development of T2DM (56). A spectrum of mechanisms could account for the inverse association between elevated HDL-C levels and diminished diabetes risk, such as anti-inflammatory responses, enhanced insulin secretion, and augmented glucose uptake by peripheral muscles (57). These insights emphasize the critical role of HDL-C in the management and prophylaxis of T2DM.

In our analysis, no significant association was identified between physical activity and the incidence of T2DM. This is similar to the findings of a Meta-analysis (58). However, the findings of some studies suggest that exercise plays a crucial role in the prevention of T2DM (59). Several potential explanations for this difference are worthy of consideration. Firstly, the characteristics of study population might have affected the observed relationship. The population in the database we used is based on a general population without diabetes, and the sample size is large. Secondly, the methodology employed to assess exercise may not have fully captured the subtleties of physical activity relevant to T2DM prevention. The reliance on self - reported measures gives rise to the possibility of recall bias, potentially resulting in an inaccurate estimation of actual physical activity. Finally, interactions between exercise and other variables, such as BMI, age, or sex, might modify the effect of exercise on T2DM risk. In the future, randomized controlled trials may be needed to further clarify the relationship between exercise and T2DM.

These considerations emphasize the complexity of studying the relationship between exercise and T2DM and underline the necessity for more in - depth research in this area. The development of a sophisticated risk model that incorporates multiple predictors and achieves an AUC of 0.90 represents a significant leap forward in the predictive analytics for T2DM. The model’s high sensitivity and specificity at a cutoff of >0.02 indicate its potential application in clinical settings for the early identification and focused intervention of T2DM. This integrated model captures the complex etiology of T2DM and carries substantial clinical significance for the prophylaxis and treatment of the condition. Ongoing research is essential to authenticate the model’s efficacy and to investigate the potential addition of other risk factors to further refine its predictive power.

While our study pioneers an in-depth eight-year exploration of T2DM incidence and its determinants within a Japanese cohort, offering unprecedented insights and a robust dataset, it is vital to acknowledge its basic shortcomings. Our predictive model, despite its potential to revolutionize primary care and facilitate early T2DM detection, does not account for the complexity introduced by uncollected variables. In the current study, we focused on the components of the metabolic syndrome as risk factors for T2DM. However, it is important to acknowledge that other potential risk factors, such as family history, medication use, and environmental/occupational exposures, were not specifically examined due to the limitations of our data. Notably, uric acid levels, which have been implicated in the pathogenesis of T2DM, were not available for analysis. This limitation should be considered when interpreting our results, as the absence of these variables may affect the comprehensiveness of our risk factor assessment. In future studies, it would be beneficial to include a more comprehensive range of risk factors to better understand their individual and combined contributions to the development of T2DM. Moreover, this data set only relies on FPG, HbA1c and self-reported to diagnose T2DM. Compared to OGTT, it may impact the incidence rate of T2DM and exclusion of T1DM or other specific types of diabetes. In addition, our study confines itself to the assessment of baseline risk factor levels, overlooking their dynamic changes over time. This static approach may underestimate the significance of temporal variations, which could significantly influence disease progression. Future longitudinal investigations should address this gap by tracking these fluctuations to provide a more comprehensive risk profile. Another limitation stems from the exclusive focus on the Japanese population, limiting the generalizability of our findings to other ethnic backgrounds. To overcome this, multi-ethnic collaborations are crucial to validate our observed risk factors and to explore potential ethnic-specific influences. Furthermore, the observational design of our study precludes definitive causal inferences, demanding meticulously planned randomized controlled experiments to confirm the impact of targeted interventions based on our identified risk factors. These trials would provide stronger evidence for the development of tailored prevention strategies.

## 5 Conclusions

The current study underscores the critical importance of adopting a comprehensive, multifactorial strategy for the prevention of T2DM. Specifically, our findings highlight the significance of early interventions aimed at modifying key risk factors, particularly obesity, unhealthy alcohol consumption, and smoking habits. Furthermore, the routine monitoring of essential metabolic indicators, such as HbA1c and liver enzymes, emerges as a fundamental component in the risk assessment and management of T2DM. By addressing these factors proactively, we can significantly enhance prevention efforts and improve health outcomes for at-risk populations.

## Data Availability

The datasets presented in this study can be found in online repositories. The names of the repository/repositories and accession number(s) can be found below: https://datadryad.org/stash/dataset/doi:10.5061/dryad.8q0p192.
